# Association between changes in genital immune markers and vaginal microbiome transitions in bacterial vaginosis

**DOI:** 10.1038/s41598-025-88208-9

**Published:** 2025-01-28

**Authors:** Philipp Foessleitner, Briah Cooley Demidkina, Wafae El-Arar, Miles Goldenberg, Meena Murthy, Agnes Bergerat, Ofri Bar, Douglas S. Kwon, Caroline M. Mitchell

**Affiliations:** 1 Vincent Center for Reproductive Biology, Massachusetts General Hospital, 55 Fruit St, Their 9, Boston, MA 02114 USA; 2 Harvard Medical School, Boston, MA USA; 3 Department of Obstetrics and Gynecology, Division of Obstetrics and Feto-Maternal Medicine, Medical University of Vienna, Vienna, Austria; 4 Department of Microbiology and Molecular Genetics, Faculty of Medicine, Hebrew University of Jerusalem, Jerusalem , Israel; 5 Ragon Institute of MGH, MIT, and Harvard, Boston, MA USA

**Keywords:** Bacterial vaginosis, Vaginal mucosal cytokines, Vaginal mucosal immune cells, Monocytes, IP-10, MIG, Bacterial infection, Urogenital diseases

## Abstract

Bacterial vaginosis (BV), characterized by an imbalance in the vaginal microbiota, is a prevalent condition among women of reproductive age and a risk factor for human immunodeficiency virus, sexually transmitted infections, and preterm birth. BV is generally considered to induce mucosal inflammation, but the specific pathways and cell types involved are not well characterized. This prospective study aimed to assess associations between microbial changes and mucosal immune responses in BV patients. Therefore, samples from 20 premenopausal women with BV and treated with metronidazole were analyzed. Vaginal swabs, menstrual cup, and endocervical cytobrush samples were collected before treatment, weekly for four weeks, and at 2, 4, and 6 months for Nugent scoring, immune cell populations and cytokine analysis. Of 105 study intervals, 27 (25.7%) showed improvement in Nugent category, 61 (58.1%) remained unchanged, and 17 (16.2%) worsened. Improvement correlated with decreased monocytes (*p* = 0.005), while worsening was linked to increased monocytes (*p* < 0.001) and dendritic cells (*p* = 0.02). B cells (*p* = 0.02) and IFN-γ-induced chemokines - IP-10 (*p* = 0.007), MIG (*p* = 0.049), and ITAC (*p* = 0.005) - were associated with improvement. In conclusion, although the T-cell-associated chemokines IP-10, ITAC, and MIG were strongly associated with improvements in Nugent category, our findings indicate that antigen-presenting cells, particularly monocytes, show the most dynamic response to shifts in the vaginal microbiota in patients with BV.

**Supplementary Information:**

The online version contains supplementary material available at 10.1038/s41598-025-88208-9.

## Introduction

Bacterial vaginosis (BV) is a common condition among women of reproductive age, characterized by an absence of lactobacilli and a diverse community of anaerobic bacteria, which often leads to increased vaginal discharge, odor, and discomfort^1,2^. Besides discomfort associated with the symptoms, BV is also associated with several adverse health outcomes presumed to be mediated by inflammation such as preterm birth^3–5^ and an increased risk of acquisition and transmission of HIV and other sexually-transmitted infections^6–8^.

The mucosal immune response in BV has been studied widely, however, study results show a great variation^9,10^. The cytokines IL-1β, IL-6, IL-8, and TNF-α are consistently found to be elevated in people with BV in cross-sectional studies, reflecting a heightened inflammatory response^10–13^. Other cytokines and chemokines such as IFN-γ, IL-1α, IL-4, IL-10, IL-17, or IL-12 demonstrate more variability in association with the microbiome across studies^10,14^. The source of these immune signaling molecules are antigen-presenting cells (APCs) such as monocytes (MC) and dendritic cells (DC) as well as the vaginal and cervical epithelium^15^. Additionally, alterations in the vaginal microbiome have been associated in some studies with increased numbers and activation of T cells, which can exacerbate inflammation and tissue damage, and increase susceptibility to HIV^9,16,17^. However, many studies are cross sectional, which limits our understanding of causality and sequence of responses to changes in the microbiome.

In this study, we aimed to assess the kinetics of mucosal immune responses to changes in the vaginal microbiota after antibiotic treatment for BV. By analyzing longitudinal samples from BV patients, we aimed to identify patterns of changes in microbiota, immune cells and cytokines that reveal causal relationships and directionality of change. By assessing these patterns, we hope to contribute to the understanding of the pathogenesis of BV and how BV influences associated risks such as preterm birth and the increased susceptibility to HIV or STIs.

## Materials and methods

### Ethics statement

The presented study was approved by the ethics committee of the Massachusetts General Hospital (Partners Institutional Review Board, IRB number: 2020P003930) and conducted in accordance with the Declaration of Helsinki and Good Scientific Practice guidelines, following the STROBE guidelines for observational studies^18^. All participants provided written informed consent.

### Setting and study population

The study was conducted at the Vincent Center for Reproductive Biology of the Massachusetts General Hospital. Eligible participants were premenopausal, assigned female at birth, aged 18–55 years, and diagnosed with bacterial vaginosis (BV) based on either Amsel criteria (assessing discharge, vaginal pH, clue cells, and odor) or Nugent score ≥ 7. Exclusion criteria included a Nugent score < 4, a recent COVID-19 infection, significant vaginal, cervical, or uterine disease such as cancer, dysplasia, other vulvovaginal infections besides BV, masculinizing hormone therapy and recent use of antibiotics, immunomodulators, or probiotics. Participants were recruited through various channels including the Mass General Brigham’s online patient recruitment portal, research recruitment letters, flyers, referrals from clinicians and advertisements placed in Massachusetts Bay Transportation Authority buses and received a compensation for study participation if enrolled.

### Study procedure and data collection

Participants attended eight study visits over a six-month period: weekly visits for the first five weeks (visits 1–5) and follow-up visits at 2, 4, and 6 months (visits 6–8). Antibiotic treatment using oral metronidazole (500 mg twice daily for 7 days) was administered between visit 1 and 2. During each visit, participants provided vaginal/endocervical swabs and used a disposable menstrual cup (Softdisc, Flex Company) for 20 min to collect vaginal fluid. A vaginal swab was used to create smears for Nugent scoring and Amsel criteria assessment. An endocervical cytobrush was collected by placing the brush in the cervical os and rotating twice. The sample was placed in R10 media (500 mL RPMI media + 5mL 200 mM L-glutamine + 5mL 1 M Hepes + 25000 IU Pencillin/ 25000 mg Streptomycin + 10% fetal calf serum) at 4 °C and processed within 2 h. Nugent criteria were assessed at each visit, Amsel criteria at inclusion and if the participant reported symptoms. If participants experienced symptoms between scheduled visits, they could attend unscheduled ad-hoc visits for additional evaluation and sample collection. If menses coincided with a scheduled visit, the visit was skipped.

### Disposable menstrual cup processing

The menstrual cup was placed in a sterile 50 ml conical tube and transported to the laboratory on ice. The sample was weighed, and the weight of a clean tube and menstrual cup subtracted to get specimen weight. The tube was centrifuged at 810xg at 4C for 10 min and then the menstrual cup removed with sterile forceps, scraping any adherent secretions into the tube with a sterile spatula. Sterile saline (500uL) was added and the vaginal fluid was homogenized by aspirating it through a blunt 16-gauge needle 20 times^19^. The sample was then aliquoted evenly into 4 cryotubes, labeled and stored frozen at -80 °C.

### Determination of Nugent score and category

Vaginal fluid was collected using sterile cotton-tipped swabs as described above and applied to a glass slide for Gram staining. Gram-stained smears were examined microscopically by two independent readers, and in cases where the two readers disagreed, a third reader was consulted. The vaginal microbiota was classified according to the Nugent scoring system^20^. This scoring system evaluates the presence or absence of *Lactobacillus spp.* (0–4 points), *Gardnerella vaginalis* (0–4 points), and *Mobiluncus spp.* (0–2 points), categorizing a score of 0–3 as normal (N), 4–6 as intermediate (INT), and 7–10 as indicative of bacterial vaginosis (BV). Additionally, the presence or absence of *Candida species* and white blood cells was assessed.

For analysis purposes, we classified intervals between two visits as either improved, unchanged, or worsened Nugent category. An improvement in Nugent category was defined as a shift from BV to intermediate (BV to INT), intermediate to normal (INT to N), or BV to normal (BV to N). Conversely, worsening intervals were defined as transitions from normal to intermediate (N to INT), intermediate to BV (INT to BV), or normal flora to BV (N to BV). Intervals where the Nugent category did not change between two consecutive visits were classified as unchanged.

### Immune cell analysis

Flow cytometry was used to evaluate alterations in the endocervical immune cell populations when the vaginal microbiota changes. Vaginal cytobrush samples were processed within 2 h of collection. Cells were stained with LIVE/DEAD fixable blue dead cell stain dye and fluorescent monoclonal antibodies specific for the following human surface markers: CD45 (Alexa700), CD8 (APC-H7), CD56 (APC), CD3 (PE-CF594), CD66b (PE), HLA-DR (FITC), CD4 (BV786), CD38 (BV650), CD14 (BV605), CD19 (BV510), CCR5 (BV421), CD11c (BUV737), and CD16 (BUV395). The samples were then fixed with 1% paraformaldehyde (PFA) and analyzed on a flow cytometer within 48 h. Flow cytometry was performed on the 4-laser LSR2 (LIVE samples) (4 L LSR2), 4-laser LSR2 (4R LSR2), and the 5-laser LSR Fortessa (5 L Fortessa) (BD). We used the following controls for each experiment: (1) an unstained sample, (2) a fluorescence minus one control for a subset of markers and (3) calibration (“rainbow”) beads. These allowed us to ensure comparability between experiments. Data analysis was conducted using FlowJo Version 7.0.0 (FlowJo Enterprise). The gating diagram is displayed in Figure S1.

### Cytokine analysis

To assess changes in the quantity of vaginal mucosal cytokines, we used quantitative fluorescent flow cytometry (Luminex^®^). Processed frozen vaginal fluid samples from the disposable menstrual cup were thawed on wet ice, and 100 uL of each sample was transferred to a 96-well plate. The samples were then diluted with 100 uL of PBS and centrifuged at 1000xg for 15 min. Afterwards, the supernatant from each well was transferred to a Millipore^®^ 0.22 μm MultiScreenHTS GV Filter Plate that was centrifuged at 2451 x g for 1 h. Cytokine quantities in the filtered supernatant samples were measured with a MILLIPLEX^®^ MAP Custom 20-Plex High Sensitivity Kit on a FlexMap 3D Luminex machine with xPONENT software. The cytokines measured in the kit were: ITAC, IFN-γ, IL-10, MIP-3α, IL-12 (p70), IL-13, IL-17, IL-1α, IL-1β, MIG, IL-21, IL-4, IL-23, IL-5, IL-6, IL-8, IP-10, MIP-1α, MIP-1β, and TNF-α. Kit reagents were diluted by a factor of 1/3, and all other steps were done according to the manufacturer’s protocol. Upper and lower limits of detection for each analyte are included in Supplemental Table 1. Samples that had analytes with bead counts below 20 were re-run. During analysis the outcome data was normalized for the menstrual cup volume.

### Statistical analysis

**Fig. 1:**
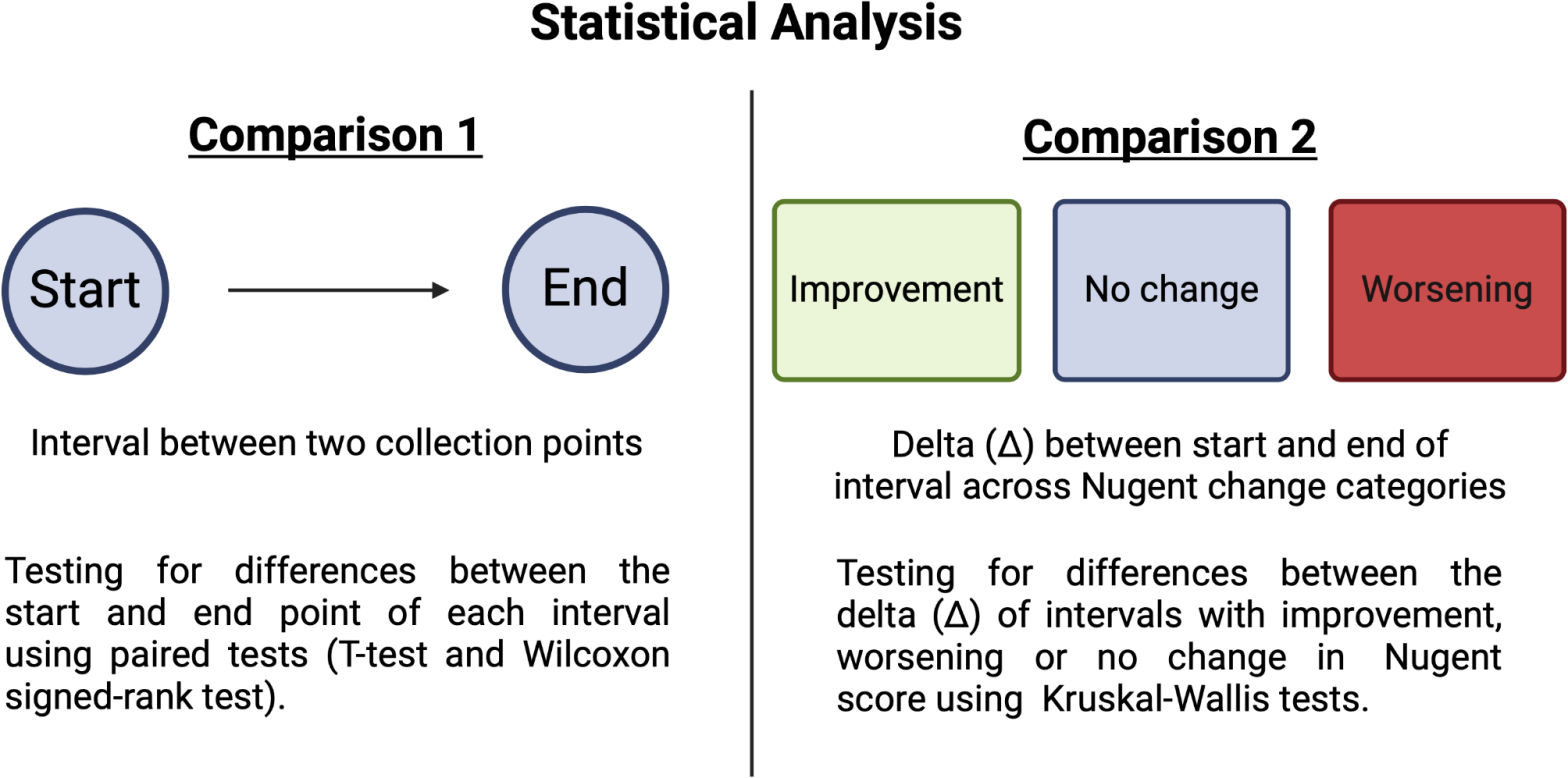
Schematic representation of the two types of comparisons used for statistical analysis of this study.

To test for changes within intervals and across Nugent categories, we conducted two main comparisons. *Comparison 1* is a paired analysis, comparing the distribution of immune cells and cytokine levels in the vaginal fluid between two consecutive collection points. For this comparison, data pairs were categorized into improved, unchanged, and worsened intervals. Depending on the normality of the data, either the paired T-test or Wilcoxon signed-rank test were applied. *Comparison 2* is an unpaired analysis that focused on differences between Nugent score change categories (improvement, no change, and worsening) in the distribution of change in immune cells and cytokine levels in the vaginal fluid. We first calculated the difference (delta, Δ) of the respective variable between two consecutive fluid collection points. Differences in the median or mean delta between the groups of improvement, worsening, or no change in Nugent category were then compared using Kruskal-Wallis tests or ANOVA depending on normality. In cases of significant results, a post-hoc test was performed to identify which specific groups differed from each other, with p-values adjusted for multiple comparisons using the Bonferroni correction. The schematic of these two comparison types are graphically presented in Fig. 1. Additionally, descriptive statistics were calculated for all variables of interest. Continuous variables are either presented as mean (standard deviation) or median [interquartile range], unless otherwise specified. Ordinally scaled and binary variables are presented with numbers and percentages. The significance level for all calculations was set to α = 0.05. Statistical analysis was performed using R Studio, Version 4.3.3.

## Results

### Study participants and baseline characteristics

A total of 33 women with suspected BV were screened for study inclusion, of whom 20 patients fulfilled the inclusion criteria and were enrolled. Three participants ended the study early, 1 after V6 due to pregnancy, 1 after V6 and 1 after V5 because they enrolled in another research study on BV treatment. Seven participants missed one visit, either due to menses or scheduling conflicts; three participants missed 2 visits, and one participant missed 3 visits. Two participants attended V7-V9, but samples were not able to be analyzed. There was only one ad-hoc visit for symptoms, at which the participant did not have BV, and which was excluded from this analysis. The mean age at inclusion was 31.3 years (standard deviation, SD, ± 8.4). Out of our 20 participants, 9 women were Black (45%), 7 White (35%), 2 Asian (10%) and 2 self-identified as other racial background (10%). Of these, 4 participants (20%) reported identifying as Hispanic. Regular menses were reported by 13/20 participants (65%) and 18 women (90%) had a history of prior episodes of BV. Additionally, 9/20 women (45%) reported a history of at least one other STI. Regarding sexual attraction, 13/20 participants (65%) stated to be attracted to the males, the remaining 7 women (35%) were attracted to both the males and females. No severe health problems were present in any participant. The characteristics of the study population are shown in Table 1.

**Table 1:** Baseline characteristics of 20 included women with BV.

Baseline characteristic		Study population (*N* = 20)
Age at inclusion, years		31.3 (± 8.4)
Patient race	Asian	2 (10.0%)
	Black	9 (45.0%)
	White	7 (35.0%)
	Other	2 (10.0%)
Education	High school	1 (50%)
	Unfinished college	4 (20%)
	College degree	10 (50%)
	Graduate degree	5 (25%)
Sexual orientation	Heterosexual	13 (65%)
	Bisexual	6 (30%)
	Other	1 (5%)
Birth control	Oral contraceptive pills	4 (20%)
	Copper IUD	5 (25%)
	Levonorgestrel IUD	2 (10%)
	Etonorgestrel implant	1 (5%)
	None of the above	8 (40%)
Regular menses	Yes	13 (65%)
	No	7 (35%)
Nugent score at inclusion		7 [6–8]
Prior BV	Yes	18 (90%)
Number of BV episodes in the past year (*N* = 16)	2 or less	10 (62.5%)
	3 or more	6 (37.5%)
Previous diagnosis of	Chlamydia	9 (45%)
	Gonorrhea	2 (10%)
	Trichomonas	4 (20%)
	Herpes	4 (20%)
	Abnormal Pap smear	3 (15%)

Data are presented as number (percentage), mean (± standard deviation) or median [interquartile range].

BV, bacterial vaginosis.

**Fig. 2:**
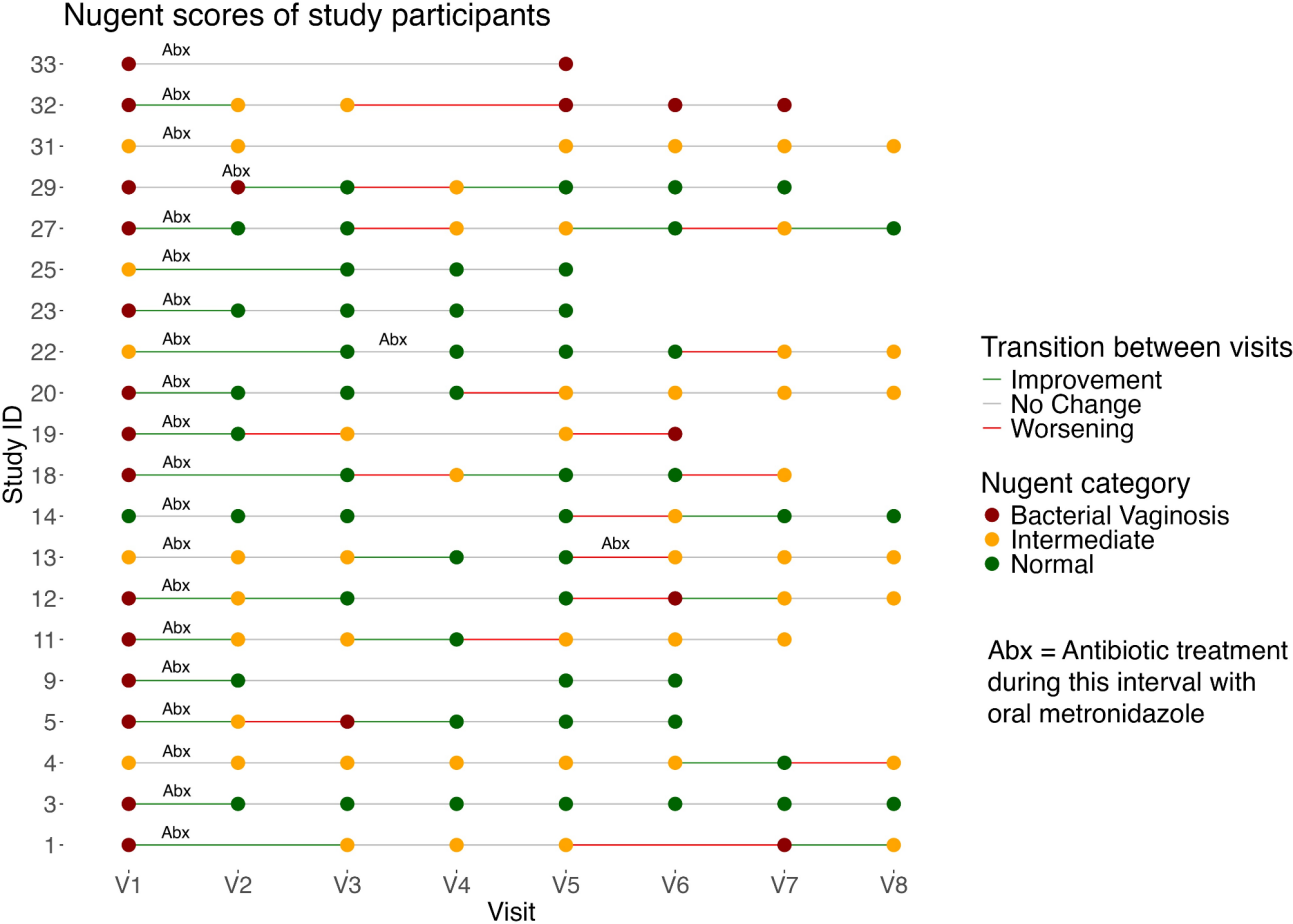
Longitudinal trajectories of the Nugent category (bullets) and its changes (lines) over time for each study patient. Antibiotic treatment was administered between Visit 1 (V1) and Visit 2 (V2).

All participants were diagnosed with BV at inclusion, either by Amsel criteria (6/20 participants) or Nugent score ≥ 7 (14/20 participants). The median Nugent score at the inclusion visit prior to metronidazole treatment (V1) was 7 [interquartile range, IQR, 6–8]. All participants completed their antibiotic treatment course, based on self-report. There were two intervals where participants reported having taken antibiotics prescribed by their own provider. Out of 105 intervals, 27 (25.7%) had improvement of the Nugent category, 17 (16.2%) a worsening of Nugent category and 61 (58.1%) unchanged Nugent category. At the first visit after antibiotic treatment, 14/20 patients (70%) improved in Nugent category and 6 patients (30%) remained in the same Nugent category. In the further course of the study, 14/20 patients (70%) experienced a worsening in Nugent category over at least one interval. The longitudinal trajectories of Nugent categories for each patient are depicted in Fig. 2, and intervals with antibiotic treatment are noted.

### Changes in vaginal mucosal immune cell populations

To analyze changes in the vaginal mucosal immune cell populations, we conducted two different comparisons. At first, we analyzed differences in the distribution of immune cells in the vaginal fluid between the start and end of an interval (*Comparison 1*). In intervals where the Nugent category improved, we observed that the median proportion of CD19 + CD45 + B-cells increased significantly from 5.3% [2.5–18.0] at the start to 13.4% [5.4–29.2] at the end of the interval (*p* = 0.02). Conversely, median monocyte (MC[HLA-DR + CD14 + CD11c+]CD45+) levels decreased from 38.1% [24.1–50.3] to 28.7% [3.0–34.8] (*p* = 0.005). In intervals with a worsening Nugent category, monocytes showed a significant increase from 10.8% [1.9–24.9] to 31.0% [16.5–31.7] (*p* = 0.009), and dendritic cells increased from 2.1% [0.8–4.7] to 4.0% [1.5–8.9] (*p* = 0.02), while CD8 + CD45 + T-cells decreased from 10.4% [3.2–14.2] to 5.6% [1.6–8.1] (*p* = 0.009). Table 2 shows the results of *Comparison 1*. Figure S2 graphically presents the significant changes in vaginal immune cell proportions within intervals of the Nugent categories.

**Table 2:** Differences (median [IQR]) in the proportion of immune cells and T cell activation markers in the vaginal fluid between the start and end of intervals with improvement, no change or worsening of Nugent category in women with BV treated with metronidazole (*comparison* 1).

Cell population	Improvement of Nugent category			No change in Nugent category			Worsening of Nugent category		
	Start	End	*P* - Value	Start	End	*P* - Value	Start	End	*P* - Value
B cells (CD19CD45+)	5.3% [2.5–18.0]	13.4% [5.4–29.2]	**0.02**	10.7% [4.6–24.2]	11.2% [4.7–23.1]	0.64	16.6% [10.8–31.2]	10.7% [4.0–27.5]	0.38
Dendritic cells (DCsCD45+)	4.5% [3.5–9.7]	3.8% [1.6–9.5]	0.69	5.1% [3.5–9.5]	4.7% [3.4–7.7]	0.49	2.1% [0.8–4.7]	4.0% [1.5–8.9]	**0.02**
Monocytes (MCsCD45+)	38.1% [24.1–50.3]	28.7% [3.0–34.8]	**0.005**	27.8% [15.0–35.2]	24.5% [14.9–42.8]	0.75	10.8% [1.9–24.9]	31.0% [16.5–31.7]	**0.009**
CD4 + T cells (CD4 + CD45+)	13.8% [10.6–16.8]	13.4% [8.1–19.6]	0.99	14.4% [8.5–19.6]	12.8% [8.9–19.4]	0.58	11.6% [7.9–23.6]	8.5% [4.4–12.9]	0.13
CD8 + T cells (CD8 + CD45+)	8.8% [6.2–12.3]	8.9% [4.6–11.5]	0.68	8.9% [5.4–11.6]	7.9% [4.2–12.2]	1.0	10.4% [3.2–14.2]	5.6% [1.6–8.1]	**0.009**
CD38 cells (CD38 + CD4+)	29.5% [18.4–40.3]	25.6% [18.0–43.3]	0.90	26.3% [18.2–43.9]	35.3% [24.7–48.4]	**0.03**	40.3% [25.0–50.8]	21.3% [9.7–33.2]	**0.03**
CCR5 cells (CCR5 + CD4+)	50.4% [21.4–65.9]	47.0% [30.0–62.3]	0.92	33.9% [20.5–51.7]	46.6% [27.2–65.7]	0.90	65.9% [44.5–75.5]	47.1% [20.5–63.3]	**0.03**
HLA-DR cells (HLA-DR + CD4+)	7.4% [4.8–14.5]	7.0% [4.5–14.8]	0.87	7.5% [4.1–18.4]	11.8% [6.8–21.5]	**0.04**	18.4% [11.1–22.6]	9.3% [4.6–23.6]	0.13
CD38 cells (CD38 + CD8+)	29.0% [12.1–35.3]	23.4% [10.0–47.7]	0.92	23.8% [16.6–40.2]	35.4% [23.1–52.3]	**0.01**	39.5% [13.3–63.1]	19.6% [7.4–29.0]	**0.03**
CCR5 cells (CCR5 + CD8+)	57.6% [26.7–70.3]	53.5% [34.3–73.6]	0.87	48.7% [32.7–69.4]	59.6% [36.1–79.1]	0.18	71.2% [47.4–82.9]	57.3% [38.7–68.6]	0.10
HLA-DR cells (HLA-DR + CD8+)	9.3% [4.5–19.5]	7.4% [3.2–25.9]	0.97	8.6% [4.3–15.9]	14.6% [7.1–24.3]	**0.008**	11.7% [6.5–24.9]	10.7% [4.6–18.1]	0.71

Significant values are in bold.

In the second analysis, we examined the changes (delta) in the proportion of immune cells between two consecutive visits across different Nugent score categories (improvement, no change, and worsening) (*Comparison 2*). In intervals where the Nugent category improved, the median delta for CD19 + CD45 + B-cells was + 5.7% [0.7–25.0] compared to intervals with no change (-0.3%, [-5.3–5.2]) or worsening (-1.7%, [-8.6–3.3]) in Nugent category (*p* = 0.02). The median monocyte delta (MCsCD45+) was a decrease of -14.7% [-27.4 – -0.03] in intervals with an improvement in Nugent category and a significant increase of + 12.6% [4.5–34.1] in intervals with worsening in Nugent category (*p* < 0.001). The detailed results of the changes in immune cell proportions across Nugent score categories are presented in Table 3.

**Table 3:** Changes (delta; displayed as median [IQR]) in the proportion of immune cells between two consecutive visits across different Nugent categories (improvement, no change, and worsening) in women with BV treated with metronidazole (*comparison 2*).

Cell population	Improvement of Nugent category	No change in Nugent category	Worsening of Nugent category	*P* - Value
B cells (CD19CD45+)	+ 5.7% [0.7–25.0]	-0.3% [-5.3–5.2]	-1.7% [-8.6–3.3]	**0.02**
Dendritic cells (DCsCD45+)	-0.6% [-4.8–3.0]	-0.2% [-4.1–2.3]	+ 2.0% [0.5–3.8]	0.09
Monocytes (MCsCD45+)	-14.7% [-27.4 – -0.03]	-1.3% [-9.6–12.5]	+ 12.6% [4.5–34.1]	**< 0.001**
CD4 + T cells (CD4 + CD45+)	+ 1.9% [-0-5–4.8]	+ 1.2% [-5.5–7.3]	-3.7% [-9.8–1.8]	0.24
CD8 + T cells (CD8 + CD45+)	-0.5% [-4.5–3.3]	+ 0.5% [-3.8–3.3]	-2.9% [-7.4 – -1.1]	0.07
CD38 cells (CD38 + CD4+)	+/-0% [-7.1–10.3]	+ 6.2% [-8.4–29.4]	-22.0% [-31.4 – -4.9]	**0.008**
CCR5 cells (CCR5 + CD4+)	-0.6% [-16.7–15.7]	+ 4.6% [-6.9–28.2]	-20.2% [-31,4 – -7.4]	**0.01**
HLA-DR cells (HLA-DR + CD4+)	-0.2% [-5.3–5.3]	+ 3.6% [-3.3–11.9]	-4.6% [-16.8–3.0]	**0.04**
CD38 cells (CD38 + CD8+)	+ 0.2% [-16.0–19.9]	+ 9.6% [-2.0–31.1]	-16.7% [-46.3 – -0.1]	**0.005**
CCR5 cells (CCR5 + CD8+)	+/-0% [-12.1–14.2]	+ 2.7% [-15.8–25.0]	-15.4% [-37.2 – -4.2]	0.08
HLA-DR cells (HLA-DR + CD8+)	-0.2% [-4.7–4.6]	+ 2.5% [-1.2–12.8]	-5.1% [-13.4–11.3]	0.15

Significant values are in bold.

Additionally, we analyzed changes in expression of T cell activation markers CCR5, HLA-DR and CD38. Between the start and end of intervals with worsening in Nugent category, we saw a significant decrease in proportions of CCR5 + CD4 + and CD38 + CD4 + T cells from 65.9% [44.5–75.5] to 47.1% [20.5–63.3] (*p* = 0.03) and from 40.3% [25.0–50.8] to 21.3% [9.7–33.2], respectively (*p* = 0.03) (*Comparison 1*). In CD8 + T cells this pattern was only observed for the CD38 activation marker (39.5% [13.3–63.1] to 19.6% [7.4–29.0], *p* = 0.03). No significant changes were observed in intervals with improvement of Nugent category. Interestingly, we found significant increases of CD4 + T cell activation markers CD38 and HLA-DR in intervals with no change in Nugent category from 26.3% [18.2–43.9] to 35.3% [24.7–48.4] (*p* = 0.03) and from 7.5% [4.1–18.4] to 11.8% [6.8–21.5], respectively (*p* = 0.04). Similar changes were observed in proportions of CD38 + CD8 + T cells, which increased from 23.8% [16.6–40.2] to 35.4% [23.1–52.3] (*p* = 0.01) and HLA-DR + CD8 + cells from 8.6% [4.3–15.9] to 14.6% [7.1–24.2] (*p* = 0.008). The Nugent score in these intervals, however, did not change (median change: 0 [-1–1]). All activation marker results of *Comparison 1* are presented in Table 2. Across Nugent categories (*Comparison 2*), we observed significantly greater decreases of the proportion of CCR5+, CD38 + and HLA-DR + CD4 + T cell activation markers in intervals with worsening in Nugent category compared to intervals with no change (*p* = 0.01, *p* = 0.008 and *p* = 0.04, respectively) as well as a greater decrease in the proportion of CD38 + CD8 + T cell activation marker in the same interval (*p* = 0.005). Detailed results are presented in Table 3.

### Changes in mucosal cytokine levels

For the analysis of change in mucosal cytokine population in the vagina after antibiotic treatment for BV, we applied the same statistical principles used for assessing immune cell populations. First, we compared cytokine levels at the beginning and end of intervals with improvement, no change or worsening of Nugent category (*Comparison 1).* We found that interferon-γ induced chemokines, IP-10, MIG and ITAC significantly increased in intervals with improvement in Nugent category. Specifically, IP-10 increased from 6.2 pg/ml vaginal fluid [4.8–7.1] to 7.5 pg/ml [6.2–8.4] (*p* = 0.01); MIG rose from 7.9 pg/ml [6.0–9.4] to 9.1 pg/ml [7.9–10.5] (*p* = 0.04) and ITAC elevated from 2.5 pg/ml [1.6–3.5] to 3.5 pg/ml [2.4–4.1] (*p* = 0.007). Other analyzed cytokines, including the pro-inflammatory markers IL-1ɑ, IL-1β, IL-6 and IL-8 and also interferon-γ (IFN-γ), showed no significant changes across intervals of improvement, no change, or worsening in Nugent category. Detailed results are presented in Table 4. The cytokine levels for each patient are presented as heatmap in Figure S3.

**Table 4:** Differences (median [IQR]) in the cytokine levels (pg/ml) in the vaginal fluid between the start and end of intervals with improvement, no change or worsening of Nugent category in women with BV treated with metronidazole (*comparison 1*).

Cytokine population	Improvement of Nugent category			No change in Nugent category			Worsening of Nugent category		
	Start (pg/ml)	End (pg/ml)	*P* - Value	Start (pg/ml)	End (pg/ml)	*P* - Value	Start (pg/ml)	End (pg/ml)	*P* - Value
IL-1α	6.8 [6.2–7.8]	6.7 [6.0–7.1]	0.12	6.7 [6.0–7.2]	6.8 [6.3–7.2]	0.20	7.0 [6.1–7.5]	6.9 [6.5–7.4]	1.00
IL-1β	4.8 [2.8–7.0]	3.7 [2.5–5.6]	0.23	4.6 [2.6–6.3]	4.6 [2.5–6.6]	0.50	5.1 [2.5–6.1]	5.3 [3.0–6.7]	0.96
IL-4	2.4 [1.6–2.8]	2.7 [1.6–3.1]	0.44	2.7 [2.2–3.3]	2.7 [2.3–3.3]	0.79	2.7 [1.6–3.3]	2.5 [1.6–2.7]	0.61
IL-5	-0.02 [-0.2–0.3]	0.1 [-0.2–0.3]	0.49	0.1 [-0.2–0.3]	0.3 [-0.2–0.3]	0.52	0.3 [-0.2–1.1]	0.1 [-0.2–0.3]	0.31
IL-6	4.1 [1.1–6.4]	3.7 [2.5–7.7]	0.50	5.5 [2.9–7.7]	4.8 [2.7–7.1]	0.59	3.8 [2.0–5.8]	4.1 [1.4–5.2]	0.71
IL-8	8.5 [6.9–9.2]	8.7 [7.4–9.2]	0.85	8.9 [8.0–9.3]	8.9 [8.1–9.5]	0.57	8.4 [7.9–9.1]	8.4 [7.1–9.3]	0.62
IL-10	1.5 [1.1–2.3]	1.4 [1.2–2.1]	0.82	1.8 [1.4–2.6]	1.6 [1.4–2.6]	0.38	1.4 [1.2–2.3]	1.6 [1.4–2.2]	0.80
IL-12 (p70)	-0.02 [-0.2–0.3]	0.1 [-0.2–0.3]	0.85	0.1 [-0.2–0.3]	0.1 [-0.2–0.3]	0.68	0.1 [-0.2–0.3]	0.1 [-0.2–0.3]	0.35
IL-13	0.9 [0.2–1.6]	0.9 [0.3–1.6]	0.87	1.4 [0.3–1.7]	1.1 [0.3–1.8]	0.77	0.9 [0.5–1.2]	1.2 [0.8–1.7]	0.80
IL-17	2.1 [1.3–2.9]	1.8 [1.0–2.7]	1.00	2.3 [1.6–2.9]	2.4 [1.8–3.0]	0.53	1.7 [0.9–3.5]	2.2 [1.4–2.8]	0.86
IL-21	-0.02 [-0.3–0.8]	0.3 [-0.4–0.8]	0.82	0.3 [-0.4–0.6]	0.3 [-0.4–0.8]	0.38	0.5 [-0.2–1.3]	0.3 [-0.1–0.8]	0.13
IL-23	3.4 [3.1–4.0]	3.5 [3.2–3.7]	0.87	3.6 [3.3–3.8]	3.5 [3.1–4.1]	0.81	3.7 [3.3–4.5]	3.6 [3.1–3.8]	0.17
IFN-γ	2.0 [1.2–2.5]	2.1 [1.2–3.0]	0.42	2.0 [1.2–3.0]	2.0 [1.2–2.8]	0.87	1.8 [1.2–2.5]	2.3 [1.2–2.8]	0.93
IP-10	6.2 [4.8–7.1]	7.5 [6.2–8.4]	**0.01**	7.3 [6.3–8.3]	7.1 [6.3–8.2]	0.77	7.4 [6.4–8.5]	7.0 [5.5–7.8]	0.10
MIG	7.9 [6.0–9.4]	9.1 [7.9–10.5]	**0.04**	9.3 [7.6–10.6]	9.6 [8.0–10.6]	0.23	8.9 [8.2–10.5]	8.4 [7.4–10.3]	0.17
ITAC	2.5 [1.6–3.5]	3.5 [2.4–4.1]	**0.01**	3.4 [2.5–4.5]	3.1 [2.4–4.2]	0.30	3.2 [2.5–3.9]	2.9 [2.5–3.7]	0.38
MIP-1b	3.0 [1.7–5.2]	2.8 [1.6–3.7]	0.43	3.1 [1.8–5.0]	3.3 [1.7–5.1]	1.00	3.3 [1.7–3.5]	2.4 [1.7–3.2]	0.55
MIP-3a	4.1 [3.1–5.1]	4.6 [3.3–5.6]	0.06	5.3 [3.9–6.4]	4.9 [3.9–6.6]	0.68	4.4 [3.4–6.2]	4.9 [3.0–5.4]	0.32
TNF- α	0.8 [-0.3–2.8]	0.1 [-0.0–0.9]	0.32	0.1 [-0.2–1.4]	0.1 [-0.3–1.0]	0.62	0.1 [-0.3–1.0]	0.1 [0.1–1.0]	0.88

Significant values are in bold.

**Fig. 3:**
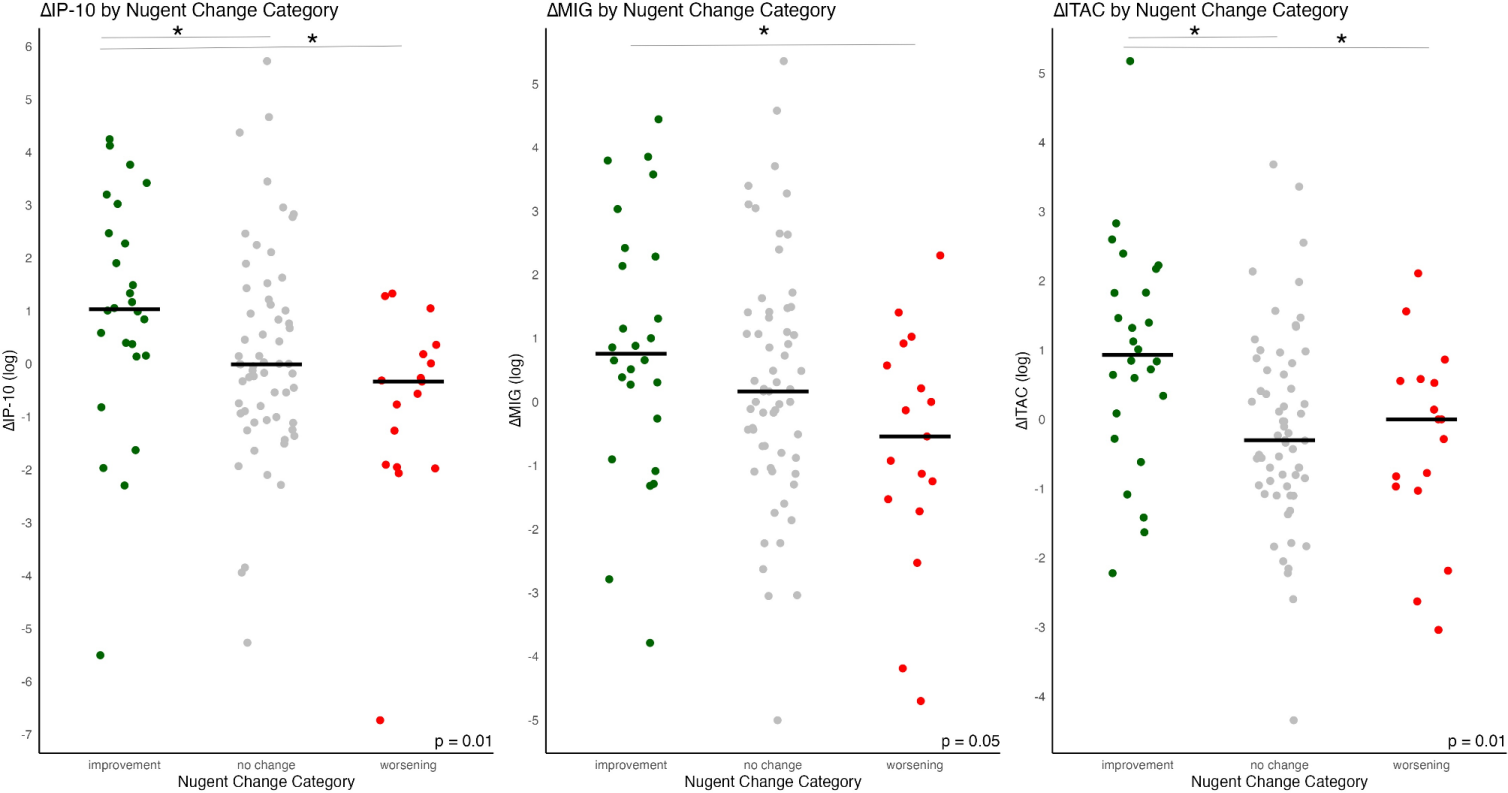
Changes of IFN-γ induced chemokine levels, IP-10, MIG and ITAC across intervals with improvement, no change and worsening in Nugent category after antibiotic treatment for BV using Kruskal-Wallis tests (*Comparison 2*). Significant post-hoc comparisons between groups are noted with a line and asterix. Data are presented log-transformed for better comparability.

Applying *Comparison 2*, we analyzed the changes in cytokine concentrations across intervals with improvement, no change and worsening in Nugent category. Significant changes were observed in the same IFN-γ induced chemokines that showed significance in *Comparison 1*. IP-10 had a significantly different delta in intervals with improvement by a median of + 1.0 pg/ml vaginal fluid [0.2–2.4], compared to intervals with no change (-0.009 pg/ml, [-1.0–1.1]) and worsening (-0.3 pg/ml, [-1.9–0.2]) of Nugent category (*p* = 0.007). For MIG, *Comparison 2* revealed a significant increase in improving vs. worsening intervals (+ 0.8 pg/ml [-0.1–2.3] vs. -0.5 pg/ml [-1.5–0.6]; *p* = 0.049). The ITAC concentrations rose by a median + 0.9 pg/ml vaginal fluid [0.2–1.8] in intervals with improvement in Nugent category, compared to intervals with no change (-0.3 pg/ml [-1.0–0.7]) and worsening (0pg/ml [-1.0–0.6]) (*p* = 0.005). These changes are presented in Fig. 3. Detailed results for cytokine comparisons across Nugent categories are provided in Table 5, while Figure S4 graphically represents the changes in cytokines levels across the three intervals.

**Table 5:** Changes (delta; displayed as median [IQR]) in the cytokine levels (pg/ml) between two consecutive visits across different Nugent categories (improvement, no change, and worsening) in women with BV treated with metronidazole (*comparison 2*).

Cell population	Improvement of Nugent category	No change in Nugent category	Worsening of Nugent category	*P* - Value
IL-1α	-0.2 [-1.1–0.3]	+ 0.08 [-0.2–0.7]	+ 0.3 [-0.9–0.7]	0.15
IL-1β	-0.4 [-2.2–1.4]	+ 0.3 [-0.5–1.0]	-0.4 [-1.3–2.0]	0.48
IL-4	+/-0 [-1.0–1.2]	0 [-0.6–0.6]	-0.2 [-0.9–0.3]	0.66
IL-5	0 [-0.2–0.6]	0 [-0.2–0.3]	0 [-0.4–0.2]	0.48
IL-6	+ 0.1 [-1.8–3.0]	-0.03 [-1.0–0.8]	+ 0.8 [-1.0–1.9]	0.57
IL-8	-0.1 [-0.7–0.7]	0 [-0.5–0.6]	-0.01 [-1.1–0.3]	0.66
IL-10	0 [-0.6–0.8]	0 [-0.9–0.4]	0 [-0.4–0.6]	0.77
IL-12 (p70)	0 [-0.2–0.5]	0 [-0.2–0.2]	0 [-0.4–0.2]	0.63
IL-13	+ 0.1 [-0.9–0.9]	0 [-0.7–0.9]	+ 0.2 [-0.8–0.7]	0.99
IL-17	-0.2 [-0.8–1.1]	+ 0.05 [-0.6–0.8]	+ 0.2 [-1.2–0.7]	0.88
IL-21	+ 0.2 [-0.3–1.0]	-0.03 [-0.4–0.6]	0 [-1.1–0.2]	0.29
IL-23	-0.08 [-0.3–0.7]	0 [-0.5–0.7]	-0.3 [-1.1–0.2]	0.33
IFN-γ	+ 0.3 [-0.9–1.2]	0 [-0.5–0.8]	0 [-1.1–0.8]	0.77
IP-10	+ 1.0 [0.2–2.4]	-0.01 [-1.0–1.1]	-0.3 [-1.9–0.2]	**0.007**
MIG	+ 0.8 [-0.1–2.3]	+ 0.2 [-0.8–1.4]	-0.5 [-1.5–0.6]	**0.049**
ITAC	+ 0.9 [0.2–1.8]	-0.3 [-1.0–0.7]	0 [-1.0–0.6]	**0.005**
MIP-1b	-0.2 [-2.1–1.1]	0 [-0.9–0.8]	-0.4 [-0.9–0.7]	0.67
MIP-3a	+ 0.3 [-0.1–1.2]	-0.03 [-0.8–0.6]	-0.1 [-0.6–0.7]	0.26
TNF- α	-0.2 [-1.2–0.8]	0 [-0.7–0.8]	0 [-0.8–0.6]	0.67

Significant values are in bold.

## Discussion

In this study, we investigated the kinetics of mucosal immune responses in women with BV during and after antibiotic treatment, with a focus on the association between changes in vaginal microbiota, immune cell populations, and cytokine levels. We found that an improvement in Nugent category was associated with an increase in endocervical CD19 + B-cells and a decrease in endocervical monocytes, accompanied by an increase of IFN-γ induced chemokines, IP-10, MIG and ITAC on the cytokine level. A worsening Nugent category was associated with an increase in antigen-presenting cells, specifically monocytes and dendritic cells, and a decrease in CD8 + T cells. No significant changes in cytokine concentrations were observed in intervals with a worsening Nugent category.

Monocytes and dendritic cells are antigen presenting cells which produce cytokines that activate T cells^9^. In an African cohort, endocervical antigen presenting cells were the population with the most differentially expressed genes between people with *Lactobacillus*-dominated vaginal microbial communities vs. highly diverse communities^15^. A study by Qulu et al.^21^. , which examined the mucosal immune environment in BV patients before and at 6 and 12 weeks after metronidazole treatment, reported decrease in proportion of endocervical monocytes among women who were cured. Our findings align with this evidence, showing a significant decrease in monocytes during intervals where the Nugent category improved. The Qulu study also found a significant increase in CD38 + CD4 + T cells in women with persistent BV after treatment^21^. In contrast, our study observed a reduction in CD4 + T cell activation markers CD38 + and CCR5 + over intervals where the Nugent category worsened. However, we did not see a difference in the pattern of change in CD4 + T cells across intervals of Nugent score improvement, no change, or worsening. Notably, we assessed cellular populations at 7 timepoints, whereas the study by Qulu et al.^21^. assessed immune dynamics only twice post-treatment. Future longitudinal studies are needed to confirm APC and T cell dynamics after changes in the vaginal microbial community. Additionally, the possibility that changes in T cell activation could impact the microbial community should be evaluated.

We observed a significant association between a decrease in Nugent score and increased levels of the IFN-γ induced chemokines IP-10, MIG, and ITAC, all of which target the CXCR3 receptor on T cells. Our findings align with recent studies showing increased IP-10 and MIG levels after resolution of BV following antibiotic treatment^22–24^. In vitro, a mixture of short-chain fatty acids representative of metabolites from BV-communities suppressed epithelial cell production of IP-10 after stimulation with the toll-like receptor agonists Poly-IC or Pam^25^. Additionally, treating polarized vaginal epithelium in vitro with *G. vaginalis* decreases production of IP-10, suggesting that during BV there may be suppression of some immune responses^26^.

In a recent randomized trial, abundance of both *L. crispatus* and *L. iners* – the most common species of vaginal lactobacilli – were positively correlated with concentration of vaginal fluid IP-10^27^. A separate study of Kenyan women found that communities dominated by *Gardnerella* subtype A, as well as highly diverse communities, were associated with lower concentrations of IP-10^28^. However, these associations seems paradoxical, as multiple studies have shown that people with the highest concentrations of vaginal fluid IP-10 and MIG are at increased risk for HIV acquisition^27,29,30^, while people with *Lactobacillus*-dominant communities have a lower risk^31^. Additionally, cross sectional studies have found decreased IP-10 to be a sensitive marker of bacterial vaginosis and sexually transmitted infections^14,32^.

Although the three analytes associated with change in Nugent category are all induced by IFN-γ, and all target a receptor most commonly found on T cells, which might explain an association with HIV risk, we did not see a difference in IFN-γ concentrations, nor in most T-cell populations between intervals with an increase vs. decrease in Nugent scores. This may suggest that the increase in IP-10, MIG and ITAC after clearance of BV is a rebound after removal of the suppressive effect of BV-associated microbes rather than an active stimulation by IFN- γ, and may be insufficient to alter HIV susceptibility.

### Strengths and limitations

A strength of our study is the characterization of both cellular and soluble mucosal immune responses during and after antibiotic treatment for BV at multiple intervals, enabling a precise characterization of the immunologic changes following therapy. This longitudinal data allows us to better assess directionality of change in each component and infer potential causal relationships. Our study population has diverse representation of self-identified race. However, our study also has limitations. The sample size, consisting of 105 intervals, was relatively small, potentially limiting the generalizability of our findings. Our study cohort was ethnically diverse, reflecting the U.S. population; however, the small sample size precluded subgroup analyses by race. Given known differences in vaginal microbiome composition among racial groups, it is possible that changes in the vaginal microbial community are associated with different patterns of immune response by race/ethnicity, which we did not have the power to detect. As race is a social construct, this could be due to social or environmental stressors, or epigenetic changes. Finally, our approach treated intervals as independent, which may not fully account for within-subject correlations or differences in the length of time between intervals. Future studies should consider mixed-effects models to address repeated measures more comprehensively and incorporate time-weighted analyses to account for variability in interval length.

## Conclusion

While the T-cell-associated chemokines IP-10, ITAC, and MIG were closely linked to improvement in Nugent score, our findings suggest that antigen-presenting cells, particularly monocytes, show the most dynamic response to shifts in the vaginal microbiota of BV patients. The significant decrease in monocytes during intervals of Nugent score improvement, along with the increase in both monocytes and dendritic cells during intervals of worsening Nugent category indicates that antigen-presenting cells rather than T cells may play a more direct role in the initial immune response to bacterial vaginosis. Further research is needed to clarify these immune dynamics and their implications for BV recurrence or clearance and HIV susceptibility.

## Electronic supplementary material

Below is the link to the electronic supplementary material.

Supplementary Material 1

## Data Availability

Data is available upon reasonable request to the corresponding author.

## Abbreviations

APCs: Antigen-presenting cells
BV: Bacterial vaginosis
CD: Cluster of differentiation
DC: Dendritic cells
HIV: Human immunodeficiency virus
IFN: γ-Interferon-gamma
IL: Interleukin
IP: 10-Interferon gamma-induced protein 10
ITAC: Interferon-inducible T-cell alpha chemoattractant
L. crispatus: Lactobacillus crispatus
L. iners: Lactobacillus iners
MC: Monocytes
MIG: Monokine induced by interferon-gamma
STI: Sexually transmitted infection
TNF: α-Tumor necrosis factor-alpha
