# Supplementary material for: Association between changes in genital immune markers and vaginal microbiome transitions in bacterial vaginosis
Source: Sci Rep. 2025 Jan 28;15:3536. doi: 10.1038/s41598-025-88208-9 (PMC11775339; doi:10.1038/s41598-025-88208-9)
Supplement: Supplementary file 1 — Supplementary Material 1 [file 41598_2025_88208_MOESM1_ESM.docx]

**Supporting Information**

________________________________________________________________

**Association between Changes in Genital Immune Markers and Vaginal Microbiome Transitions in Bacterial Vaginosis**

Philipp FOESSLEITNER^1,2,3^, Briah COOLEY DEMIDKINA^1^, Wafae EL-ARAR^1^, Miles GOLDENBERG^1^, Meena MURTHY^1^, Agnes BERGERAT^1^, Ofri BAR^1,2,4^, Douglas S. KWON^2,5^, Caroline M. MITCHELL^1,2^

^1^Vincent Center for Reproductive Biology, Massachusetts General Hospital, Boston, MA, USA

^2^Harvard Medical School, Boston, MA, USA

^3^Department of Obstetrics and Gynecology, Division of Obstetrics and Feto-Maternal Medicine, Medical University of Vienna, Vienna, Austria

^4^ Department of Microbiology and Molecular Genetics, Faculty of Medicine, Hebrew University of Jerusalem

^5^Ragon Institute of MGH, MIT, and Harvard, Massachusetts General Hospital, Harvard Medical School, Boston, MA, USA

**Figure S1.** (A) Gating diagram used in the presented study to differentiate between different endocervical immune cells following flow cytometry. (B) Graphical example of the gating process (Participant 25 – Visit 1).

1. **Gating diagram**

1. **Example of gating process (Participant 25 – Visit 1)**

**Figure S2.** Significant changes of vaginal immune cell populations (%cell population out of CD45+ cells) between start and end of intervals with improvement, no change or worsening in Nugent category (*Comparison 1*). The y-axis shows the different immune cell proportions of the 105 intervals with Nugent category transitions. A) Monocytes (%MCsCD45+), B) B cells (%CD19+CD45+), C) Dendritic cells (%DCsCD45+), D) CD8 T cells (%CD8+CD45+).


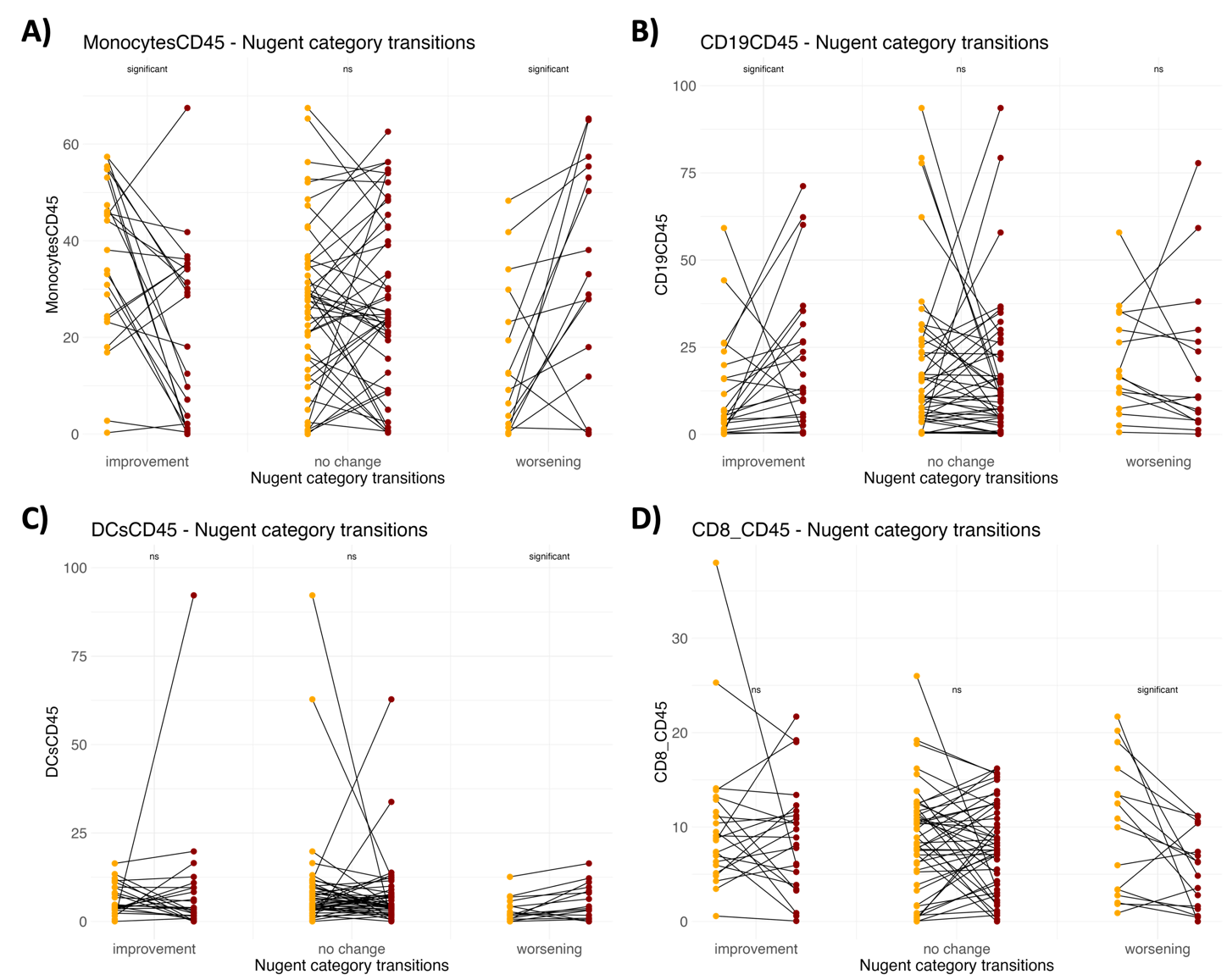


**Figure S3.** Heatmap displaying cytokine levels (pg/ml of vaginal fluid) and Nugent category for each of the 20 study participants, with values presented in a scaled and log-transformed format to enhance comparability.


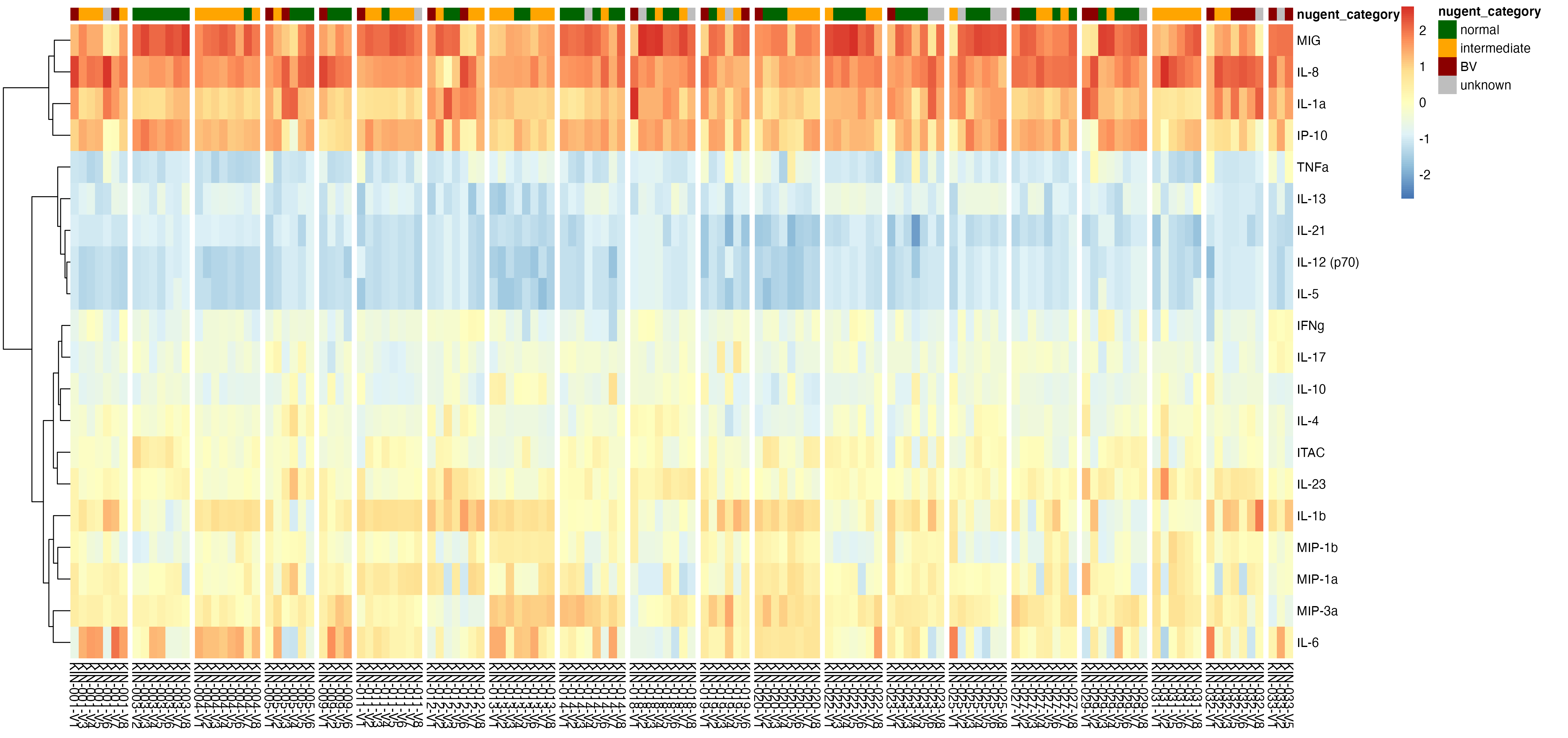


**Figure S4.** Development of cytokine levels across intervals with improvement, no change and worsening in Nugent category after antibiotic treatment for BV (*Comparison 2*). Data are presented log-transformed for better comparability.


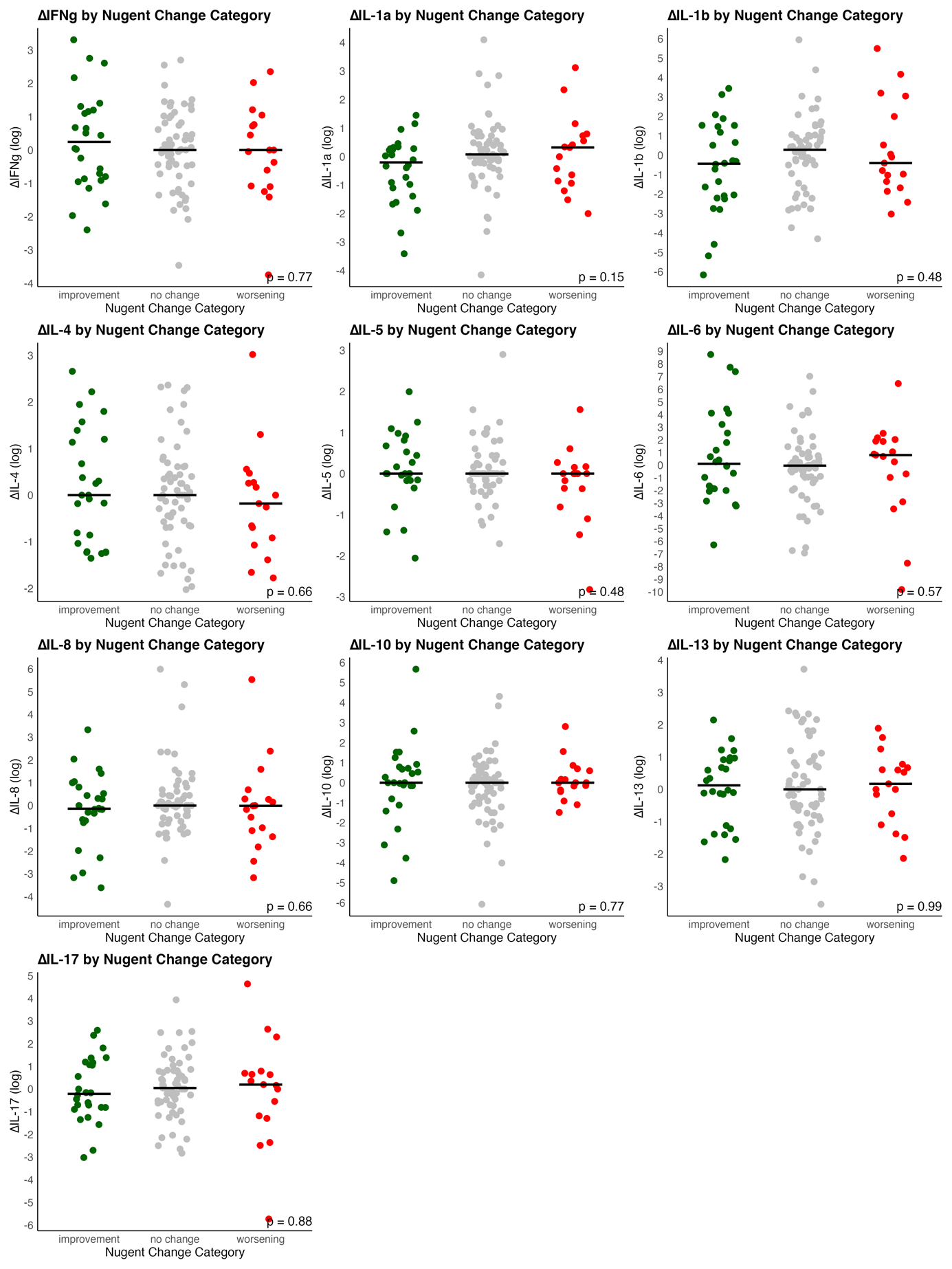


**Continuation Figure S4.** Development of cytokine levels across intervals with improvement, no change and worsening in Nugent category after antibiotic treatment for BV (*Comparison 2*). Data are presented log-transformed for better comparability.  **
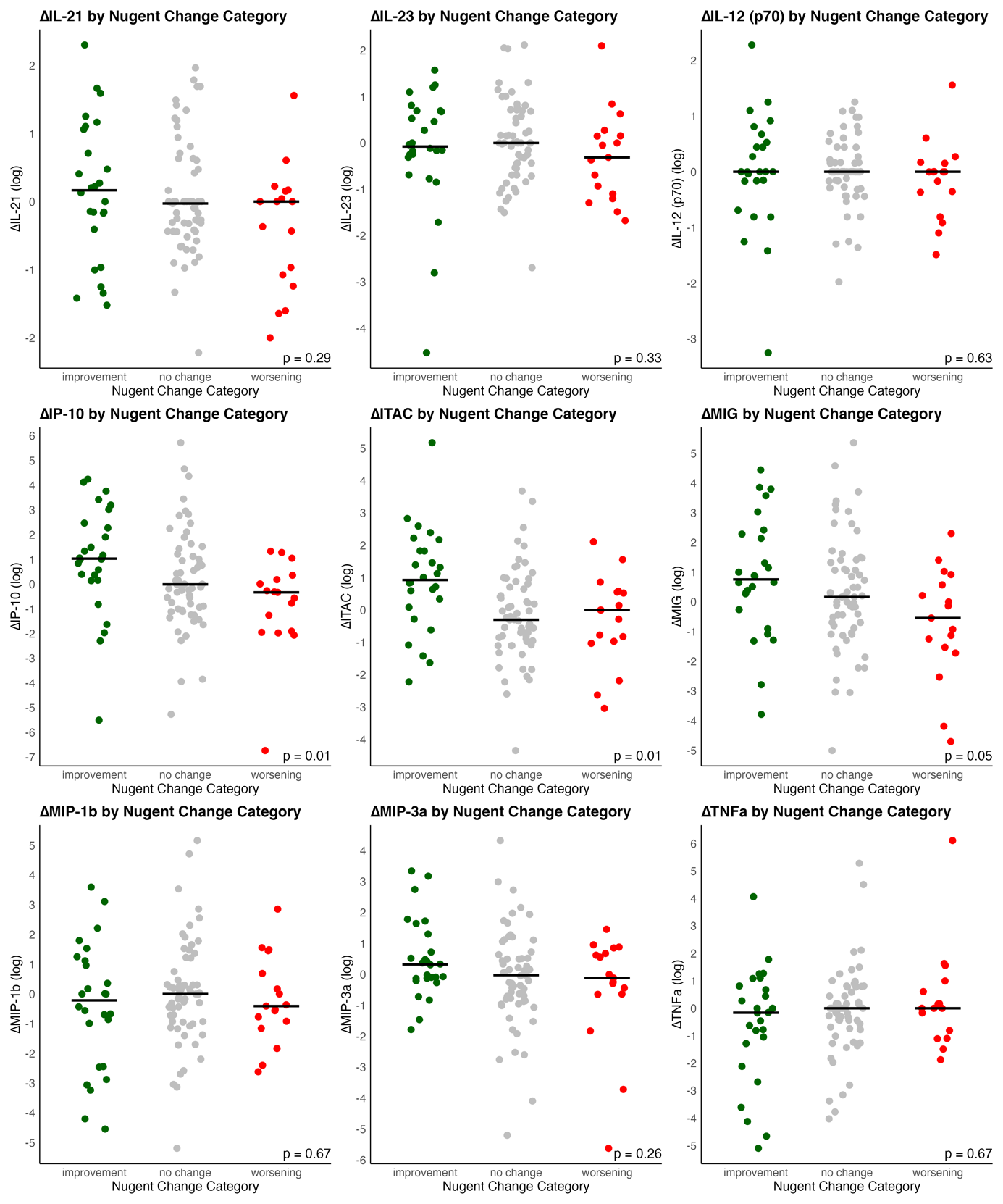
**

**Supplemental Table 1: Lower limit of detection for Luminex analytes**

| Analyte | Lower (pg/mL) | Upper (pg/mL) |
| --- | --- | --- |
| IL-6 | 0.18 | 750 |
| IL-13 | 0.24 | 1000 |
| IL-21 | 0.24 | 1000 |
| IL-8 | 0.31 | 1250 |
| MIP-1a | 0.31 | 1250 |
| TNFa | 0.43 | 1750 |
| IL-12 (p70) | 0.49 | 2000 |
| IL-1b | 0.49 | 2000 |
| IL-5 | 0.49 | 2000 |
| IFNg | 0.61 | 2500 |
| MIP-3a | 0.61 | 2500 |
| IL-1a | 0.61 | 2500 |
| IP-10 | 0.61 | 2500 |
| IL-17 | 0.73 | 3000 |
| MIP-1b | 0.92 | 3750 |
| ITAC | 1.46 | 6000 |
| IL-10 | 1.46 | 6000 |
| IL-4 | 1.83 | 7500 |
| MIG | 3.05 | 12500 |
| IL-23 | 7.93 | 32500 |
